# Burden of influenza among hospitalized febrile children in Ghana

**DOI:** 10.1111/irv.12507

**Published:** 2017-10-27

**Authors:** Benedikt Hogan, Luise Ammer, Marlow Zimmermann, Tabea Binger, Ralf Krumkamp, Nimako Sarpong, Theresa Rettig, Denise Dekker, Benno Kreuels, Lisa Reigl, Kennedy G. Boahen, Charity Wiafe, Yaw Adu‐Sarkodie, Ellis Owusu‐Dabo, Jürgen May, Daniel Eibach

**Affiliations:** ^1^ Infectious Disease Epidemiology Bernhard Nocht Institute for Tropical Medicine Hamburg Hamburg Germany; ^2^ German Center for Infection Research (DZIF) Hamburg‐Borstel‐Lübeck Germany; ^3^ College of Health Sciences Kumasi Centre for Collaborative Research in Tropical Medicine (KCCR) KNUST Kumasi Ghana; ^4^ Department of Child Health Agogo Presbyterian Hospital Agogo Ghana; ^5^ Division of Tropical Medicine I. Department of Internal Medicine University Medical Centre Hamburg‐Eppendorf (UKE) Hamburg Germany; ^6^ Department of Clinical Microbiology Kwame Nkrumah University of Science and Technology (KNUST) Kumasi Ghana; ^7^ Department of Global Health School of Public Health College of Health Sciences KNUST Kumasi Ghana

**Keywords:** Africa, bacteremia, children, fever, influenza, malaria

## Abstract

**Background:**

Influenza surveillance data from Africa indicate a substantial disease burden with high mortality. However, local influenza data from district hospitals with limited laboratory facilities are still scarce.

**Objectives:**

To identify the frequency and seasonal distribution of influenza among hospitalized febrile children in a rural hospital in Ghana and to describe differential diagnoses to other severe febrile infections.

**Methods:**

Between January 2014 and April 2015, all children with a temperature of ≥38°C admitted to a district hospital in Ghana were screened for influenza A and B by RT‐PCR and differentiated to subtypes A(H1N1)pdm09 and A(H3N2). Malaria microscopy and blood cultures were performed for each patient.

**Results:**

A total of 1063 children with a median age of 2 years (IQR: 1‐4 years) were recruited. Of those, 271 (21%) were classified as severe acute respiratory infection (SARI) and 47 (4%) were positive for influenza, namely 26 (55%) influenza B, 15 (32%) A(H1N1)pdm09, and 6 (13%) A(H3N2) cases. Influenza predominantly occurred in children aged 3‐5 years and was more frequently detected in the major rainy season (OR = 2.9; 95% CI: 1.47‐6.19) during the first half of the year. Two (4%) and seven (15%) influenza‐positive children were co‐diagnosed with an invasive bloodstream infection or malaria, respectively.

**Conclusion:**

Influenza contributes substantially to the burden of hospitalized febrile children in Ghana being strongly dependent on age and corresponds with the major rainy season during the first half‐year.

## 1 INTRODUCTION

Globally influenza has long been regarded as a major public health concern. Not only the elderly but also very young children have been identified as a vulnerable group for influenza infections. A recent meta‐analysis estimated 90 million cases of influenza and 20 million episodes of influenza‐associated acute lower respiratory infections (ALRI) annually worldwide in children below 5 years.^1^ As a result, influenza is the second most common pathogen identified in children with ALRI after respiratory syncytial virus (RSV) and followed by parainfluenza virus.^2^ About 870 000 annual hospitalizations in children below 5 years of age have been attributed to influenza with influenza‐associated hospitalization rates being three times higher in developing than in industrialized countries.^3^ It has been assumed that 99% of inpatient deaths from severe acute respiratory infections (SARI)^4^ and 70% of all deaths attributable to influenza‐like illnesses (ILI) among children below 5 years of age occurred in developing countries.^5^

The extent of influenza infections in Africa is now slowly being recognized due to strengthened national influenza surveillance systems across the continent.^6^ Most countries have had inadequate data on the influenza disease burden until the influenza A(H1N1) pandemic in 2009. Since then, many countries implemented hospital‐based influenza surveillance among SARI patients.^3^, ^7^

In Ghana, ILI sentinel surveillance within outpatient facilities across all regions was established in 2007, which screened 2357 samples in 2014 (https://www.ghanahealthservice.org). The burden of influenza disease among hospitalized children in Ghana has so far only been studied in large tertiary hospitals.^8^, ^9^ However, data on the burden of influenza are particularly important for healthcare workers in small rural or district hospitals with no or limited laboratory facilities, considering that influenza infections are not easily distinguishable from other febrile infections such as malaria and may therefore lead to false treatment decisions.^10^

This study aims to identify the proportion and seasonal distribution of influenza infections among hospitalized febrile children in a rural district hospital in Ghana to inform healthcare workers on the contribution of influenza as a differential diagnosis to other severe febrile infections.

## 2 METHODS

### 2.1 Study site and sample collection

Study participants were recruited at the pediatric ward of the Agogo Presbyterian Hospital (APH), a district hospital with 250 beds, situated in the Asante Akim North municipality of the Ashanti Region in Ghana. The climate is tropical with two rainy seasons from March to June and from September to October.^11^ The study area is located in a holoendemic malaria region with perennial malaria transmission.

Oropharyngeal swabs (Copan, Italy) were taken from all children aged between 1 month and 15 years with a tympanic temperature of ≥38°C between January 2014 and April 2015. Swabs were taken at admission and immediately transferred in viral transport medium and stored at −20° until RNA extraction. For each patient, two independent slide readers conducted malaria microscopy on Giemsa‐stained thick and thin smears and a blood culture was performed on standard media (Oxoid, Basingstoke, UK). The following respiratory signs and symptoms were assessed by the study physician: abnormal lung auscultation, breathing difficulties, chest indrawing, chest pain, coryza, cough, intercostal retractions, nasal flaring, sore throat, and stridor. Repeated visits of study children were considered as new visits if they were at least 30 days apart. SARI was diagnosed according to the World Health Organization (WHO) case definition in a hospitalized patient with fever of ≥38°C or history of fever, with cough and an onset of illness within the last 10 days.^12^

### 2.2 Sample processing and virus detection

RNA was extracted from oropharyngeal swabs with the RTP Pathogen Kit (Stratec biomedical, Birkenfeld, Germany) and eluted in 120 μL. Screening of samples for influenza A and B was conducted with the RealStar^®^ Influenza RT‐PCR Kit 1.0 (Altona Diagnostics, Hamburg, Germany) as described by the manufacturer with half the reaction volume. Positive influenza A samples were further differentiated for A(H1N1)pdm09 and A(H3N2) following the real‐time RT‐PCR protocol recommended by the WHO.^13^ PCRs were performed in 25 μL using the Superscript^®^ III OneStep RT‐PCR with Platinum Taq DNA Polymerase (Invitrogen, Karlsruhe, Germany) containing 5.0 μL RNA template, 5.5 μL water, 12.5 μL 2× reaction buffer, 40.0 μmol/L of each forward (A(H3N2): 5′‐AGCAAAGCCTACAGCAA‐3′, A(H1N1)pdm09: 5′‐GAGCTAAGAGAGCAATTGA‐3′) and reverse (A(H3N2): 5′‐GACCTAAGGGAGGCATAA‐3′, A(H1N1)pdm09: 5′‐GTAGATGGATGGTGAATG‐3′) primer, 10.0 μmol/L probe (H3N2: 5′‐Fam‐CCGGCACATCATAAGGGTAACA 3′‐BHQ‐1, A(H1N1)pdm09: 5′Fam ‐TTGCTGAGCTTTGGGTATGA ‐3′‐BHQ‐1), and 0.5 μL enzyme. Conditions for the reverse transcription PCR were 50°C for 30 minutes, followed by 2 minutes of initial denaturation at 95°C and 45 cycles at 95°C for 15 seconds and 55°C for 30 seconds.

### 2.3 Epidemiological analysis

Categorical variables were described as frequencies and percentages and continuous variables as medians and their corresponding interquartile ranges (IQRs). All data analyses were performed with Stata 14 (StataCorp LP, College Station, TX, USA).

Children were grouped by age into the categories <3, 3‐5, and >5 years. To quantify the association between a given exposure and outcome, odds ratios (OR) with their respective 95% confidence intervals (CI) were calculated.

### 2.4 Ethical considerations

The Committee on Human Research, Publications and Ethics, School of Medical Sciences, Kwame Nkrumah University of Science and Technology, Kumasi, Ghana and the “Ethikkommission der Ärztekammer Hamburg,” Germany provided ethical approvals for this study. All participants were informed about the study's purpose and procedures. Written informed consent was obtained from the parents or the guardian on behalf of the study children prior to study enrollment.

## 3 RESULTS

A total of 1063 hospital visits were made from 991 study children. Sex distribution was slightly unbalanced with 586 male cases (55%) aged 0‐14 years (median: 2 years, IQR: 1‐4 years) and 477 female cases (45%) aged 0‐13 years (median: 2 years, IQR: 1‐4 years). At recruitment, the children presented with a median temperature of 39.0°C (IQR: 38.5‐39.6°C).

During the study period, 47 (4%) children were diagnosed with influenza, of which 21 (45%) tested positive for influenza A and 26 (55%) for influenza B. Influenza A cases were divided into the virus subtypes A(H1N1)pdm09 (n = 15; 71%) and A(H3N2) (n = 6; 29%).

In total, 274 (26%) children were diagnosed with SARI, which was predominantly found in the age group <3 years (194/618; 31%), with a median of 2 years (IQR: 1‐3) (Table 1). Among SARI patients, 21 (8%) were positive for influenza while 26 (3%) of the non‐SARI patients had influenza (OR = 2.4; 95% CI: 1.34‐4.42). In contrast to SARI cases, influenza patients had a median age of 3 years (IQR: 1‐5) and were most frequently detected in the 3‐5 years age group (n = 18; 6%) with an odds ratio of 2.0 (95% CI 1.0‐3.9) (Table 1).

**Table 1:** Influenza and SARI cases stratified by sex and age

		Influenza		SARI	
		n (%)	OR (CI)	n (%)	OR (CI)
Age group					
<3	(n = 618)	22 (4)	1 (N.A.)^a^	194 (31)	1 (N.A.)^a^
3‐5	(n = 292)	18 (6)	2.0 (1.04‐3.9)	58 (20)	0.6 (0.4‐0.7)
>5	(n = 164)	7 (4)	1.5 (0.6‐3.5)	22 (13)	0.3 (0.2‐0.54)
Sex					
Male	(n = 591)	20 (3)	1 (N.A.)^a^	153 (26)	1 (N.A.)^a^
Female	(n = 483)	27 (6)	1.7 (0.3‐3.08)	121 (25)	1 (0.76‐1.33)

a Not available (reference group).

OR, odds ratio; CI, confidence interval; SARI, severe acute respiratory infection.

**Figure 1:**
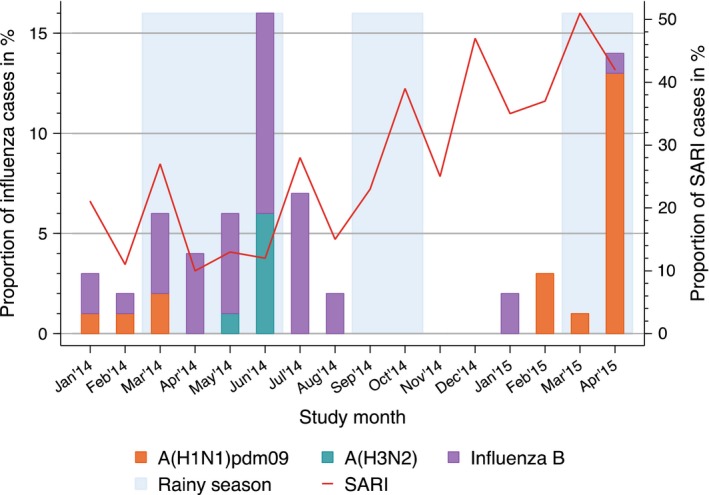
Influenza and Severe acute respiratory infection (SARI) cases per study month. Proportions of both influenza and SARI cases were calculated using the total number of recruited patients per study month. Influenza was further defined on subtype level: namely influenza B, influenza A(H1N1)pdm09, and influenza A(H3N2)

All influenza‐positive children were observed during the period between January and August 2014, with a peak in June, as well as January to April 2015 (last study month) with a peak in April (Figure 1). The observed influenza seasons partially overlap with the major annual rainy season (OR = 2.9; 95% CI: 1.47‐6.19) of the study area (Figure 1). However, no influenza activity was detected in the months September to December 2014, including the short second rainy period in September and October. The SARI cases seem to have no association with the rainy season (OR = 1.2; 95% CI: 0.09‐1.57) (Figure 1). Three different influenza subtypes circulated in the 2014 influenza season, with influenza B causing the majority (71%; 24/34) of cases, followed by A(H3N2) (18%; 6/34) and A(H1N1)pdm09 (9%; 3/34). In 2015, influenza was caused by the influenza strains A(H1N1)pdm09 (85%; 11/13) and influenza B (15%; 2/13).

Among influenza‐positive children, cough was the most common respiratory symptom (45%; n = 21), followed by coryza (19%; n = 9) and chest indrawing (9%; n = 4). At least one respiratory symptom was observed among 45% (n = 21) of influenza patients. Influenza‐positive and influenza‐negative children showed no differences concerning tympanic temperature (OR: 1.1, CI: 0.7‐1.63). The median duration of hospitalization was 2 days (IQR of 2‐4 days) and none of the children died during their stay.

Among influenza cases, seven (15%) had concomitant malaria with a median parasitemia of 12 231/μL (IQR: 8 606/μL‐35 599/μL). The detection of influenza was negatively associated with malaria disease (OR = 0.1; 95% CI: 0.05‐0.25). Concomitant invasive bacterial bloodstream infections with *Salmonella enterica* were diagnosed in two influenza‐positive children (4%).

Socioeconomic data showed that patients had 1‐13 siblings (median: 3, IQR: 2‐4) and 2‐22 people living together per household (median: 6, IQR: 4‐7). Neither the number of siblings nor the household size showed a statistically relevant relationship toward whether a case had influenza or not (data not displayed).

## 4 DISCUSSION

Within our study group, SARI cases made up more than a quarter of all febrile pediatric hospital admissions. Of the SARI cases, 8% are diagnosed with influenza A/B, which is in line with a large African multicountry surveillance project, which identified influenza in 5%‐26% of SARI cases.^6^ From Ghana, similar SARI rates (8%‐9%) have been reported.^8^, ^14^ Although the SARI case definition showed a good association with influenza in this study, it is noteworthy that influenza was still identified in 3% of non‐SARI cases. It has been shown before that influenza case definitions are highly age‐dependant and performances vary due to the unspecific clinical picture of influenza.^15^ Hence, laboratory‐based surveillance systems using a SARI definition will probably underestimate the true burden of influenza infections.

The median age of children with influenza was higher than the median age of SARI cases. This age distribution is probably due to the high frequency of other serious respiratory pathogens, notably respiratory syncytial virus (RSV), among very young children.^16^The outpatient department of the same study hospital reports human parainfluenza viruses 1‐4, enteroviruses and adenoviruses as the predominant respiratory pathogens in children below 5 years of age, while influenza A/B was the most frequently detected respiratory virus in older children (5‐12 years).^11^ However, in the present study, samples were not tested for other respiratory viruses; therefore, this assumption could not be further investigated. The higher age of children with influenza at the outpatient department compared to hospitalized children in the present study can be explained by the more severe disease presentation among young children, which is illustrated by high death rates in this age group.^3^ Influenza‐related deaths were not reported during the present study, although high case fatality ratios, more than seventeen times as high as in industrialized countries, were estimated for developing countries.^1^ This study's data are in line with findings from three Ghanaian tertiary hospitals, which also did not report any influenza‐associated deaths during a 30 months surveillance period.^8^ However, these findings may be biased by poor healthcare access, leading to an underestimation of the influenza disease burden including case fatalities.

Compared to data from the Global Influenza Surveillance and Response System (GISRS; http://www.who.int/influenza/gisrs_laboratory/en/), this study shows a similar pattern, in which influenza activity prevails end of a year/beginning of the next, until early/midsummer, which corresponds to the major rainy season. However, in contrast to outpatient‐based studies from Ghana, no influenza viruses were detected from September to December, which comprises the smaller second annual rainy season.^11^, ^14^ Other studies confirmed that influenza seasonality in countries near the equator is roughly correlated to rainfall, but less pronounced than in southern African countries such as Zambia, South Africa, and Madagascar, where influenza corresponded with the drier, cooler winter months of June to August.^17^, ^18^ GISRS data for Ghana show a similar influenza subtype distribution for 2014 and 2015, despite the fact that subtype A(H3N2) was not detected in 2015 in this study. However, the study period ended in April 2015; therefore, data for 2015 should be interpreted with caution.

Clinically, influenza is difficult to distinguish from other tropical diseases, in particular malaria, which temporally coincides during the rainy seasons.^19^ Interestingly, results of this study show that children hospitalized with influenza were less likely to have malaria than those without influenza. This effect is caused by hospital admission dynamics, meaning that children with a severe infection, such as influenza, have a reduced likelihood of another severe concomitant febrile illness (eg, malaria) being the reason for their hospital admission. This effect, also known as “Berkson's bias,” can also be observed for concomitant influenza/bacterial bloodstream infections, which are very infrequent in this study.^20^ The use of rapid diagnostic influenza tests in the study setting would help to distinguish influenza from other febrile infections and would identify the causative agent for the substantial number of hospitalized SARI cases, which otherwise remain undiagnosed. However, rapid diagnostic influenza tests have shown low sensitivities in a recently published meta‐analysis and therefore must be interpreted with caution.^21^

This study has a few limitations. Oropharyngeal swabs were used for all patients, although the combination of nasopharyngeal and oropharyngeal swabs is reported to be more sensitive for virus detection.^22^ The study was conducted throughout a 16‐month period. Therefore, temporal patterns from this study can give a rough estimate, however, to time public health interventions, such as vaccinations, longer studies over a period of at least 2 years are required. Finally, this study aimed to assess the burden of influenza infections among hospitalized children. As hospitalization rates surely depend on the proximity to a healthcare facility and health‐seeking patterns, these findings are not suitable to estimate influenza incidences in the general population. Another limiting factor of hospital‐based surveillance is the time delay from symptom onset to specimen collection, which might underestimate influenza prevalence in patients living in very distant communities.

## 5 CONCLUSION

SARI and influenza contribute substantially to the burden of hospitalized febrile children in Ghana. In comparison with SARI, which is most frequently found in children aged <3 years, influenza predominantly occurs in children aged 3‐5 years and is associated with the major annual rainy season during the first half‐year. During this time period, the use of rapid diagnostic tests may be considered on the pediatric ward, taking into account the test's low sensitivity. Distinguishing influenza from other non‐specific febrile diseases, such as malaria and invasive bloodstream infections, could help to reduce the unnecessary application of antimicrobial and antimalarial drugs.

## CONFLICT OF INTEREST

The authors have no conflict of interest.
